# Ligand recognition by the γδ TCR and discrimination between homeostasis and stress conditions

**DOI:** 10.1038/s41423-020-0503-y

**Published:** 2020-07-24

**Authors:** Malte Deseke, Immo Prinz

**Affiliations:** Institute of Immunology, Hannover Medical School, 30625 Hannover, Germany

**Keywords:** gamma-delta TCR, ligands, antigen recognition, Gammadelta T cells, T-cell receptor

## Abstract

T lymphocytes comprise cells expressing either an αβ or a γδ TCR. The riddle how αβ TCRs are triggered by specific peptides presented in the context of MHC was elucidated some time ago. In contrast, the mechanisms that underlie antigen recognition by γδ TCRs are still baffling the scientific community. It is clear that activation of γδ TCRs does not necessarily depend on MHC antigen presentation. To date, diverse and largely host-cell-derived molecules have been identified as cognate antigens for the γδ TCR. However, for most γδ TCRs, the activating ligand is still unknown and many open questions with regard to physiological relevance and generalizable concepts remain. Especially the question of how γδ T cells can distinguish homeostatic from stress conditions via their TCR remains largely unresolved. Recent discoveries in the field might have paved the way towards a better understanding of antigen recognition by the γδ TCR and have made it conceivable to revise the current knowledge and contextualize the new findings.

## Introduction

T cells are divided into αβ and γδ T cells based on the expression of their respective T-cell receptor (TCR). With a frequency of 0.5–16% of all T cells in human peripheral blood γδ T cells are the smaller subset although higher abundances can be observed in peripheral tissues.^1,2^ Nevertheless, this enigmatic immune cell subset is conserved among almost all jawed vertebrates, which indicates their importance for the immune system. They are able to recognize a plethora of infections such as by mycobacteria,^3^ *Plasmodium*,^4,5^ or cytomegalovirus (CMV)^6^ and induce potent infection containing reactions, such as granzyme and cytokine release.^7,8^ Besides, γδ T cells were also shown to be effective in inducing antitumor responses.^9–11^

Besides the different immune receptors expressed by γδ T cells like NK cell receptors and NCRs, their TCR is one of the main cell-surface molecules that is involved in the recognition of pathological conditions. This assumption is supported by blocking experiments^12,13^ and by the clonal expansion of specific γδ TCRs upon infections.^14,15^ Like its αβ counterpart, the γδ TCR is composed of two chains, named gamma and delta, whose diversity is generated by the recombination of variable (V), diversity (D, only in δ-chain) and joining (J) fragments. With some exceptions in sharks,^16^ somatic hypermutation as in immunoglobulin genes does not occur in TCRs so that the recombined region, which makes up the complementarity defining region 3 (CDR3), comprises most of the receptor’s diversity. The recognition of antigens, however, seems to be entirely different when compared between αβ and γδ TCRs. Most αβ TCRs bind to major histocompatibility complexes (MHC) I or II presenting small peptide fragments derived from pathogens or tumor specific proteins. Together with co-receptor engagement of CD4 or CD8 and co-stimulation through CD28, this elicits T-cell activation. In contrast, the γδ TCR does not require MHC-mediated antigen presentation and no general requirement for co-receptor interaction has been identified so far. This has led to the notion that the mere binding of the γδ TCR to its cognate antigen is sufficient for activation. Moreover, γδ TCRs have the ability for both innate and adaptive ligand recognition via either germline-encoded regions of the receptor, reminiscent of PRRs or adaptive antigen binding via the CDRs.^17^

Although central for understanding γδ T-cell biology and their application in therapeutic approaches, the identification of γδ TCR antigens has been proven challenging for several reasons. First, since no general restricting molecule could be identified for the γδ TCR, the antigens could be virtually any molecule present on cell surfaces or in the surrounding extracellular space. This becomes particularly problematic if not only proteins, which are already very diverse, but also carbohydrates, lipids and nucleic acids could be recognized or at least involved in the recognition because this further increases the complexity of the question and is technically demanding to address. Second, the affinity of TCRs to their antigens is typically low being mostly within the range of 1–100 µM. Therefore, classical methods of protein biochemistry cannot be applied.^18^ Alternative methods like the generation of blocking antibodies, genetic approaches or tetramer staining with known T-cell antigens is on the one hand side tedious and labor-intensive or on the other hand requires a priori knowledge of possible candidates, which introduces a bias.^18,19^ Third, it is difficult to assess whether the recognition of certain antigens by γδ TCRs can be generalized, because a number of known antigens are bound solely by particular clones that have been identified in individuals. Moreover, findings in the mouse system can in most cases not be translated into the human system and vice versa because the TCR sequences and subsets in mice and humans differ substantially. This also complicates the assessment of physiological relevance of many human γδ TCR ligands since they can be identified only in vitro without the possibility to test their functionality in transgenic animals.

Despite all these obstacles, several antigens for the γδ TCR have been identified since the discovery of γδ T cells (see Table 1). Due to recent progress made in this field, we aim to summarize in this review the current knowledge about adaptive recognition of MHC-like molecules, the concept of immunoglobulin-like antigen recognition as well as innate recognition of phosphoantigens and butyrophilins by the γδ TCR. Moreover, we speculate on how the γδ TCR might be able to discriminate between physiological and pathological conditions.

**Table 1:** List antigens for the γδ TCR

Name	Species	TCR V-usage	Affinity	Comments	Reference
*MHC and MHC-like recognition*					
I-E^b, k, s^	Mouse	Vγ1^+^/Vγ2 ^+^	>240 µM (estimated)		^22,48,119^
H-2^k, b, f, q, s^	Mouse	Vγ2Vα11	N.D.		^30^
HLA-A24	Human	Vδ1^+^	N.D.	Allo-HLA recognition	^31^
HLA-B27-ci	Human	Vγ4Vδ1	N.D.	Allo-HLA recognition	^32^
HLA-A2	Human	Vδ1^+^	N.D.	Allo-HLA recognition	^33^
HLA-A∗24:2	Human	Vγ5Vδ1	N.D.	Allo-HLA recognition but peptide loading required for increased stability of MHC	^23^
HLA-A2/MART-1	Human	Vδ1^+^	2.9–71 µM	Response restricted to MHC-presented MART-1 peptide	^34^
CD1-d	Human/Mouse	Vδ1^+^/Vδ3^+^	16–33 µM	Affinity higher upon lipid-antigen presentation but binding also to non-presenting CD1-d	^12,24,35–37,39,40^
CD1-c	Human	Vδ1^+^	23–125 µM	Affinity higher upon lipid-antigen presentation but binding also to non-presenting CD1-c	^38^
Qa-1^b^/Glu^50^Tyr^50^	Mouse	N.D.	N.D.	Unclear, if antigen is presented or not	^41,42^
MR-1	Human	Vδ1^+^	2.7–30.6 µM	No specificity for presented antigens	^43^
EPCR	Human	Vγ4Vδ5	90 µM	Generation of blocking antibody to identify antigen	^25^
MICA	Human	Vδ1^+^	110–900 µM	High-affinity NKG2D-ligand	^10,44–46^
ULBP4	Human	Vγ9Vδ2	N.D., but direct interaction shown by ELISA	High-affinity NKG2D-ligand	^13^
T10/T22	Mouse	Diverse/clones G8 (Vγ4δ5) and KN6 (Vγ4δ10)	0.1 µM	Used for generation of γδ TCR-transgenic mice with defined specificity	^47–50^
*Ig-like recognition*					
Annexin A2	Human	Vγ8Vδ3	3 µM	Generation of blocking antibody to identify antigen	^53^
EphA2	Human	Vγ9Vδ1	N.D.	γδ TCR activation only if EphA2 is bound to ephrins on γδ T cell	^54^
hMSH2	Human	Vδ2^+^	N.D.	High-affinity NKG2D-ligand	^55^
Histidyl tRNA synthetase	Human	Vγ3Vδ2	N.D.	Cell surface exposition not shown	^56,57^
HSV-gI	Mouse	Vγ2Vδ8	N.D.	Conformational epitope at N-terminus of HSV-gI	^58^
SEA	Human	Vγ9^+^ (cytotoxic response), Vγ9^−^ (cytotoxic response and proliferation)	N.D.	Superantigen from *Staphylococcus aureus*	^59^
OXYS	Human	Vγ9Vδ2	N.D.	Superantigen from Bacillus Calmette-Guérin	^60^
DX2	Human	Vγ9Vδ2	N.D.	Superantigen from *Mycobacterium tuberculosis*	^61^
Phycoerythrin (PE)	Human, Mouse, Ruminants	Human: Vγ1Vδ1 Mouse: Vγ1^+^/Vγ4^+^ (Spleen), Vγ7^+^ (intestine)	2.69 µM (Mouse)	No physiological antigen, protein from red algae	^62^
Cy3	Mouse	Vγ1^+^/Vγ4^+^	78.2 nM	Hapten, no physiological antigen	^63^
4-hydroxy-3-nitrophenyl acetyl (NP)	Mouse	Vγ1^+^	660 nM	Hapten, no physiological antigen	^63^
Insulin peptide B:9–23	Mouse	Vγ1^+^ (without immunization), Vγ4^+^ (if immunized with peptide)	N.D.	Response idependent of APCs	^64,65^
HSP-60 peptide	Mouse	Vγ1^+^	N.D.	Peptides of mycobacterial and mammalian origin recognized	^66–68^
Peptide from Listeriolysin O	Human	N.D.	N.D.		^69^
Peptide from Tetanus toxin	Human	Vγ9Vδ2	N.D.	Presented by HLA-DRw53	^70,71^
Ig λ-chain	Human	N.D.	N.D.	Recognition if antigen is not on cell surface; presentation mechanism involved?	^72,73^
Polyanionic molecules	Mouse	Vγ1Vδ6.3	N.D.	Response independent of APCs	^74^
*B7 family-like proteins and phosphoantigen recognition*					
BTN3A1	Human	Vγ9Vδ2	N.D.	Required for phosphoantigen response, binds phosphoantigen intracellularly, no direct interaction with γδ TCR shown to date	^90,91,94^
BTN2A1	Human	Vγ9Vδ2	40–50 µM	Required for phosphoantigen response, Interaction with Vγ9-chain via HV4 and CDR2	^103,104^
Skint-1	Mouse	Vγ5Vδ1 (DETC)	N.D.	Butyrophilin-like molecule required for homing of Vγ5Vδ1^+^ DETCs, direct interaction with γδ TCR not shown	^106–108^
BTNL3	Human	Vγ4^+^	SPR: 20.7 µM, ITC: 3.5 µM	Heterodimer with BTNL8, interaction via HV4 and CDR2 of γ-chain, required probably for tissue homing and homeostasis	^113,114^
Btnl6	Mouse	Vγ7^+^	N.D.	Heterodimer with Btnl6, mouse homologue of BTNL3, interaction via HV4 and CDR2 of γ-chain, required probably for tissue homing and homeostasis	^113^

## MHC-like recognition

The paradigm of γδ T cells not being restricted to MHC is based to a large extent on the observation that γδ T cells develop normally in β2-microglobulin knockout mice whereas αβ T cells are missing due to missing positive selection in the thymus.^20,21^ Nevertheless, a considerable number of γδ TCRs have been described that are able to react to MHC or MHC-like molecules.^10,22–25^ Further attempts to investigate the influence of MHC-like ligand binding by the γδ TCR on γδ T-cell development have led to contradictory results. Schweighoffer et al. reported that γδ T cells expressing a transgenic γδ TCR (G8) reactive to the MHC-Ib molecule T10/T22 develop without the presence of their cognate antigen in β2-microglobulin knockout mice.^26^ In contrast, experiments with mice expressing another T10/T22-reactive γδ TCR (KN6)^27^ and an MHC class I-reactive γδ TCR^28^ showed that the development and maturation of γδ T cells were impaired in a β2-microglobulin knockout background. As the β2-microglobulin knockout does not completely eliminate surface expression of T10/T22, a mouse model with a more specific knockout of T10 and T22 was generated and led to the conclusion that the antigen is important for the development of γδ T cells.^29^ However, even the complete absence of the respective antigen failed to abolish the generation of at least some T22-reactive γδ T cells, which might be explained by a certain plasticity of the γδ TCR for different ligands. Thus, γδ T cells can develop without the presence of their cognate antigen but their functional maturation is heavily impaired. Moreover, nonexpanded clones reactive to MHC or MHC-like molecules likely make up only a small part of the total γδ TCR repertoire and their disappearance might not be detectable in β2-microglobulin knockout mice. Therefore, clones reactive to MHC or MHC-like molecules likely coexist next to those recognizing non-MHC molecules.

Examples of γδ TCR-ligands that are classical MHC molecules comprise murine MHC class II molecule I-E^22^ and class I molecule H-2,^30^ both found to activate cytotoxic γδ T-cell clones derived from athymic mice. In humans, HLA-24,^31^ HLA-B27,^32^ and HLA-A2^33^ were specifically activating γδ T-cell clones derived from healthy individuals that have been expanded in culture. All these identified interactions have in common that they are independent of peptide presentation by the MHC, as their activation also occurred in cell lines with peptide-loading defects. These γδ T-cell clones were therefore qualified as alloreactive. The fact that some of them are able to cross-react with some subtype of the same MHC indicates that they might even be binding to less polymorphic parts of the MHC molecule. Additionally, a further alloreactive Vγ5Vδ1^+^ TCR has recently been found to recognize HLA-A∗24:02 on cancer cells.^23^ It was shown to be dependent on peptide loading of the HLA complex but not the presentation of a specific peptide. Given the increased stability of peptide-presenting MHC molecules on cell surfaces, the authors reasoned that the random peptide presentation might be rather required for target stabilization than for specific antigen presentation. In contrast to this, Vδ1^+^ γδ T cells derived in vitro from human hematopoietic stem and progenitor cell (HSPC) were reactive to MHC HLA-A2-restricted peptide presentation of the melanoma antigen MART-1.^34^ However, the resolved structure of the interacting proteins suggests that binding of the respective γδ TCRs to MART-1 presenting MHC is less peptide-centric as compared to the interaction with a MART-1-specific αβ TCR. Hence, one might speculate that MART-1-MHC-specific activation of some γδ TCR is still different from classical αβ TCR MHC-restriction and that MART-1 could also be a specific stabilizer for the MHC that is required for proper detection by the respective γδ TCRs.

Besides classical MHC recognition some γδ TCRs are reactive towards MHC class Ib or MHC-related proteins in mice and humans. The lipid-antigen-presenting molecules CD1-c and CD1-d are amongst the best studied examples of this group.^12,35–39^ Human γδ TCRs recognizing CD1-molecules are Vδ1^+^^24^ or Vδ3^+^^40^ and they can react to several presented phospho- and glycolipids.^12,36^ However, unloaded CD1-d was also able to bind to Vδ1^+^ γδ TCRs albeit with lower affinity than if presenting lipids,^24^ which would be in-line with the mentioned alloreactivity observed in MHC-reactive γδ TCRs. Another MHC-Ib molecule that is a putative ligand for the γδ TCR is murine Qa-1^b^ presenting an artificial glutamine-tyrosine polypeptide.^41^ In addition, in vivo expansion of γδ IELs in response to *Salmonella* infection was dependent on peptide loading of Qa-1^b^.^42^ Yet, unambiguous evidence that physiological peptides bound to Qa-1^b^ are specifically recognized by γδ TCRs does not exist and a mere stabilizing function as in the case of human HLA∗24:02 cannot be excluded.^23^

Other functional interactions of γδ TCRs with MHC-like molecules do not require the presentation of antigens as is the case for MHC-related protein 1 (MR-1),^43^ endothelial protein C receptor (EPCR),^25^ MHC class I-related Chain A or B (MICA/MICB),^10,44–46^ UL16-binding protein 4 (ULBP4)^13^ and T10/T22 in mice.^47–50^ The reasons are that either the reactive γδ TCRs are binding independently of the presented antigen (MR-1), no further molecules are presented (EPCR) or the antigen-binding cleft of the respective ligand is truncated, which precludes the loading of antigen (T10/T22). Thus, overall γδ TCR recognition of classical MHC or MHC-like molecules seems to be independent of the presentation of foreign antigens, which is in contrast to αβ TCR antigen binding.

Reactivity of γδ TCRs to MHC or MHC-like molecules is largely dependent on the CDRs with a substantial focus on the CDR3δ in most cases (T10/T22, CD1-d, MART-1 HLA-A2) and the TCR-chains are commonly composed of Vδ2^−^- or Vγ9^−^Vδ2^+^ sequences. Furthermore, reactive TCRs were usually derived from particular private clones (EPCR, HLA∗24:02) that were not shared between individuals or were of low abundance in peripheral blood (MR-1, CD1, T10/T22). However, γδ TCR repertoire analysis revealed that clones of the Vδ2^−^- or Vγ9^−^Vδ2^+^ subsets can undergo rapid and sustained clonal expansion in response to e.g., CMV infection^14,15^ and MART-1-HLA-A2 reactive γδ T cells could be expanded from PBMCs in vitro.^34^ These features of MHC- and MHC-like-reactive γδ TCRs are reminiscent of the adaptive responses observed in αβ T cells, hence this type of antigen recognition in γδ TCRs was termed adaptive as has been reviewed by Willcox & Willcox^18^ as well as Davey et al.^51,52^ As a consequence, it is often difficult to judge whether the ligand-specificities observed are a general phenomenon that is particularly relevant, since most of the interactions were identified in cell culture systems in vitro and, so far, evidence for physiological relevance is still rare. On the other hand, also in αβ T cells the amount of particular antigen-specific clones is low prior to expansion and it is conceivable that antigen-naïve but potentially reactive γδ T cells present at low frequencies would expand upon antigen exposure. In fact, the EPCR-reactive LES clone (Vγ4Vδ5^+^) made up about 25% of the entire T-cell repertoire in a CMV-positive transplanted patient.^25^ In addition to the low abundance of naïve γδ T cells, it is possible that other MHC- or MHC-like reactive γδ TCRs escaped the detection by tetramer staining as in the case of CD1-d or MR-1 because the affinity for their cognate antigen was too low for flow cytometry approaches. Concerning the methodology employed for the identification of the so far investigated MHC molecules as γδ TCR ligands, it has been criticized that it relied to a large extent on previous knowledge and techniques from αβ TCRs and the detection of MHC or MHC-like molecules as γδ TCR ligands might thus not appear very surprising. Despite this technical bias in many studies published in the past, the identification of HLA∗24:02 as an antigen for the alloreactive Vγ5Vδ1^+^ γδ TCR by Kierkels et al. indicates that also approaches without preconceived ideas of putative antigen candidates can reveal MHC or MHC-like molecules as γδ TCR ligands.^23^ The question to what extent the reactivity to MHC molecules can be generalized awaits further investigation and unbiased identification of reactive γδ TCRs.

## Ig-like recognition of antigen

Adaptive or adaptive like antigen recognition by γδ TCRs is by no means limited to MHC- or MHC-like molecules as shows the wide and diverse range of cell surface or soluble molecules reported to be γδ TCR antigens. These include the cell stress-induced Annexin A2^53^ and ephrin receptor A2 (EphA2),^54^ which were recognized by Vδ2^−^ γδ T-cell clones in a TCR-dependent manner on cells that were either transformed, CMV-infected or exposed to abiotic stressors, e.g., heat. The human DNA mismatch repair protein MutS-Homologue 2 (hMSH2)^55^ is found at the cell surface of malignant cells and induces target cell killing dependent on Vδ2^+^ γδ TCRs. Furthermore, histidyl tRNA synthetase^56,57^ is recognized by a Vγ3Vδ2^+^ TCR identified in a polymyositis patient although it remains elusive how this target can be reached by the γδ TCR since no cell-surface exposition has been shown to date. In both cases the γδ TCRs were Vγ9^−^Vδ2^+^.

Foreign antigens derived from pathogenic or nonpathogenic organisms that have been reported to activate γδ TCRs comprise herpex simplex virus glycoprotein I (HSV-gI) recognized by a murine Vγ2Vδ8^+^ TCR,^58^ bacterial superantigens such as SEA, OXYS, and DX2^59–61^ and even the algal protein phycoerythrin (PE), which is a prototype B-cell antigen but can also be bound by range of different murine, ruminant as well as human γδ TCRs.^62^ Similar observations were made with the haptens Cy3 and NP.^63^ However, it is very unlikely that PE or haptens represent actual antigens under physiological conditions. Nevertheless, these studies underline the plasticity of γδ TCR target recognition and it might be used in experimental models. Strikingly, not only entire proteins but also small peptide fragments can be detected by γδ TCR without the requirement for presentation by other cells or molecules. Examples are the insulin peptide B:9–23 in mice,^64,65^ peptides derived from mycobacterial and mammalian heat shock proteins in mice and humans^66–68^ and from the *Lysteria monocytogenes* protein Listeriolysin O in humans.^69^ Other peptides, however, do not bind the TCR directly but seem to require presentation on target cells such as those derived from tetanus toxin^70,71^ and from immunoglobulin λ-chain from B-cell lymphoma.^72,73^ Recognition of these peptides, whether presented or not, was found to be γδ TCR dependent but neither were direct interactions between γδ TCR and peptides shown nor exists evidence for their physiological relevance. Recently, murine γδ NKT cells with a Vγ1Vδ6.3^+^ TCR were found to be activated constitutively when cultured in vitro. The reaction was TCR-dependent and driven by polyanions present on the treated plastic ware used for culturing the cells.^74^ Although both chains were required for the reactivity, the Vγ1-chain seemed to have a slightly higher importance with only secondary relevance of the CDR3. Interestingly, many of the aforementioned peptide-specificities of γδ TCRs mice are mediated via Vγ1^+^ TCRs. Although the peptides described are not necessarily polyanionic, reactivity for short polymeric sequences by this group of TCRs might be a common feature that is, however, probably not CDR3 dependent.

Together with the different MHC and MHC-like molecules, these examples of recognized molecules illustrate the high diversity of ligands for the γδ TCR that is in contrast to αβ TCRs, which can recognize a wide range of peptides but all in the context of the less polymorphic MHC class I and II molecules. Furthermore, comparison of the CDR3 lengths between the different adaptive immune receptors revealed that overall γδ TCRs resemble more immunoglobulins with a shorter CDR3γ and a longer CDR3δ, which is in contrast to CDR3α and CDR3β that have comparable lengths.^75^ Although recognition of antigen by VDJ-recombined adaptive immune receptors depends on more factors than the mere CDR3 length, this points to an antigen-binding mode of γδ TCRs that is substantially different from αβ TCRs. Together with the great variety of γδ TCR antigens this has led to the concept of a rather immunoglobulin-like (Ig-like) recognition of antigens by γδ TCRs.^76^ In accordance with this, similar to immunoglobulins, γδ TCRs seem to be able to recognize structural as well as sequence epitopes. Moreover, the fact that many different γδ TCRs can be specific for the same target molecule as e.g., in the case of PE, CD1-d, or MR-1 is reminiscent of polyclonal antibodies with the same target specificities but different binding modes.

Despite these striking similarities between γδ TCRs and immunoglobulins with regard to their antigen recognition properties, one should always bear in mind that fundamental differences exist. The affinity of most γδ TCRs for their antigens is low in contrast to high-affinity antibodies, which has important considerations for technical applications, such as flow cytometry or co-immunoprecipitation. Due to the high density of γδ TCR molecules on the cell surface in physiological contexts and similarly high expression of the respective ligands, the high avidity of this interaction probably circumvents the single molecule low-affinity binding.^77^ To what extent a possible Ig-like antigen recognition by γδ TCRs plays a role in vivo remains a matter of debate.

## Phosphoantigen recognition

In humans the largest subset of γδ T cells in peripheral blood express a semi-invariant TCR composed of a restricted Vγ9JP rearrangement together with a more diverse Vδ2 chain.^78^ This subset is also found in other species, e.g., in non-human primates^79,80^ or alpaca but not in rodents.^81,82^ Vγ9Vδ2^+^ TCRs recognize small non-proteogenic phosphorylated molecules termed phosphoantigens (p-Ags) in an MHC-independent way.^83^ The most potent p-Ag is (E)-4-hydroxy-3-methyl- but-2-enyl pyrophosphate (HMBPP), an intermediate of the prokaryotic non-mevalonate pathway of isoprenoid biosynthesis. Isopentenyl pyrophosphate (IPP) and dimethylallyl pyrophosphate (DMAPP) are present in both prokaryotes and eukaryotes. However, their efficiency to activate Vγ9Vδ2^+^ TCRs is lower compared to HMBPP. IPP and DMAPP can accumulate inside eukaryotic cells in response to stress situations e.g., infection or malignant transformation whereas HMBPP is produced directly by pathogens such as gram-positive bacteria, *Plasmodium* or *Toxoplasma gondii*.^84^ Besides the naturally occurring p-Ags, synthetic aminobisphosphonates as alendronate, zoledronate or pamidronate can activate Vγ9Vδ2^+^ TCRs by inhibiting the enzyme farnesyl pyrophosphate synthase in the mevalonate pathway of eukaryotic isoprenoid biosynthesis leading to an intracellular accumulation of IPP.^85,86^ Vγ9Vδ2^+^ T cells can therefore be expanded for cancer immunotherapy by administration of these aminobisphosphonate drugs as has been reviewed by Morita et al.^83^ and Legut et al.^87^ The recognition of ubiquitous microbial or stress signals by γδ TCRs is reminiscent of the PAMP-detection by pattern recognition receptors, which is supported by the semi-invariant V-usage of these γδ TCRs and rather polyclonal expansions in response to p-Ags.^88^ Therefore, Vγ9Vδ2^+^ TCR-mediated recognition of p-Ags has been termed innate-like.^84^

Despite the requirement for p-Ags for the activation of Vγ9Vδ2^+^ TCRs, these small phosphorylated moieties are not the cognate antigens binding to γδ TCRs and cell to cell contact is necessary.^89^ Especially, the protein butyrophilin 3 A1 (BTN3A1) has been shown to be essential for γδ TCR-mediated p-Ag-recognition.^90,91^ BTN3A1 belongs as the other butyrophilins to the B7 receptor family-like proteins and consists of two extracellular Ig-like domains, transmembrane and juxtamembrane domains and an intracellular B30.2 domain.^92^ After the discovery of BTN3A1 as a central mediator of p-Ag reactivity, two different models of interaction with p-Ags were proposed. Vavassori et al.^93^ first showed evidence for an antigen presentation by BTN3A1 and a direct interaction between p-Ag presenting BTN3A1 and the Vγ9Vδ2^+^ TCR. In contrast, Sandstrom et al.^94^ made the observation that the intracellular B30.2 domain of BTN3A1 binds the p-Ags in a pocket with basic residues and no direct interaction between the extracellular IgV-domain of BTN3A1 and the Vγ9Vδ2^+^ TCR was detected. Subsequent mutagenesis experiments with BTN3A1 intra- and extracellular domains revealed that the second model of intracellular p-Ag binding held true.^95^ Moreover, rodent cells expressing human BTN3A1 are not able to stimulate Vγ9Vδ2^+^ TCRs upon p-Ag exposure indicating that other mechanisms and molecules are probably involved.^94^ Thus, the current concept of BTN3A1-mediated activation of Vγ9Vδ2^+^ TCRs implies that intracellular p-Ag binding to the B30.2 domain leads to conformational changes that translate to the extracellular domain of BTN3A1 in order to be sensed indirectly by Vγ9Vδ2^+^ TCRs. In-line with this hypothesis, NMR-studies of the B30.2 domain and the juxtamembrane domain of BTN3A1 revealed conformational changes upon p-Ag binding.^96–98^ Furthermore, the cytoskeletal adaptor protein periplakin as well as the GTPase RhoB were reported to interact with the B30.2 domain and thereby assist to organize BTN3A1 membrane organization influencing Vγ9Vδ2^+^ TCR activation.^99,100^

However, BTN3A1 alone is not sufficient for p-Ag recognition by Vγ9Vδ2^+^ TCRs as the BTN3A isoforms BTN3A2 and BTN3A3 were shown to be required by the use of knock down and knockout cell lines.^99,101^ In contrast to the observation of BTN3A1 homodimers, BTN3A1 and BTN3A2 seem also to be able to form heterodimers, which enable the correct BTN3A1 localization to the cell membrane and complete functionality in terms of p-Ag reactivity. Interestingly, expression of BTN3A1 alone can lead to Vγ9Vδ2^+^ TCR activation in response to p-Ag, albeit with much lower efficiency. To which extent BTN3A homo- or heterodimers play important functions in p-Ag recognition by Vγ9Vδ2^+^ TCRs or whether interconversion between both structural arrangements can occur is still to be elucidated.

Although the evidence for BTN3A1 as the p-Ag sensing molecule and its influence on Vγ9Vδ2^+^ TCR activation is compelling, no direct interaction with the γδ TCR has been established so far. Rodent cell lines expressing transgenic human BTN3A1 were shown to require human chromosome 6 for inducing functional p-Ag reactivity in Vγ9Vδ2^+^ TCRs.^102^ Hence, another component encoded on this chromosome appeared to be essential to induce p-Ag responses. Recently, BTN2A1 has been identified by two different approaches to be this enigmatic “factor X” and to be a direct ligand for Vγ9Vδ2^+^ TCRs. Rigau et al.^103^ employed tetramerized soluble TCR staining of target cells and a genome-wide CRISPR screening to identify candidates for Vγ9Vδ2^+^ TCR ligands. At the same time, Karunakaran et al.^104^ generated radiation hybrids of Chinese Hamster Ovarian (CHO) cells containing human chromosome 6 and screening for abrogation of p-Ag reactivity led to the identification of the gene encoding BTN2A1. Both publications suggested binding of the Vγ9-chain to BTN2A1 via germline-encoded regions and without major involvement of the CDRs with an affinity of around 45–50 µM. BTN2A1 itself seems to form homodimers linked by disulfide bridges and it associates with BTN3A1 on the cell surface as has been shown by co-immunoprecipitation^104^ and FRET.^103^ Interestingly, not only the extracellular IgV domains of BTN2A1 and BTN3A1 but also their intracellular regions seem to be at least in close proximity although only the B30.2 domain of BTN3A1 is able to bind p-Ag as shown by isothermal calorimetry (ITC).^94,103^ The domains of the Vγ9-chain involved in interaction with BTN2A1 were determined by mutagenesis of the γδ TCR and BTN2A1 and molecular modeling in silico. The data suggest that interaction occurs between the C, C’, F, and G β strands (CFG interface) of BTN2A1 and residues of the germline-encoded hypervariable region 4 (HV4) as well as CDR2γ.^104^ The results from the mutagenesis study conducted by Rigau et al.^103^ were not entirely consistent with these observations. Based on their mutagenesis experiments, they concluded that the outer face of the ABED β-sheet is important for the interaction with BTN2A1. However, in both cases the interaction interface of the Vγ9-chain with BTN2A1 was germline-encoded and a central glutamic acid residue at position 70 was considered to be relevant by both groups. Further investigation might be required to completely solve this discrepancy.

A still unsolved question concerning BTN-mediated p-Ag-reactivity is how the CDR3γ and CDR3δ, which are both reported to be required for a Vγ9Vδ2^+^ TCR response to p-Ag, are involved in this process.^105^ Mutations in the CDR3γ and the CDR2δ led furthermore to the abrogation of p-Ag reactivity but not the binding of the Vγ9-chain to BTN2A1. This indicated that binding to BTN2A1 is required but not sufficient for a Vγ9Vδ2^+^ TCR-mediated response.^103^ Thus, it has been speculated that at least a second interaction is necessary. Whether this is mediated by BTN3A1 as proposed by Rigau et al.^103^ or if a yet completely unknown ligand is involved as suggested by Karunakaran et al.,^104^ remains to be defined.

## The role of other B7 receptor family-like proteins

The role of butyrophilins in γδ T-cell biology exceeds their implication in p-Ag sensing and the activation of Vγ9Vδ2^+^ TCRs. Other proteins with structural similarity to the B7 receptor superfamily such as Skint-1 and Butyrophilin-like (Btnl)1 and 6 in mice as well as BTNL3 and 8 in humans were shown to be relevant for the development and possibly for the tissue homing and homeostasis of certain γδ T-cell subsets as has been reviewed recently by Haday and Vantourout.^17^

Murine DETCs bearing a canonical Vγ5Vδ1^+^ TCR require Skint-1 expression, since mice with a mutation in the *Skint-1* gene leading to a premature insertion of a stop-codon lack this skin-resident γδ T-cell subset.^106,107^ It is in particular the homing to the skin as well as the phenotype of these Vγ5Vδ1^+^ T cells that is affected rather than their differentiation in general.^108^ This effect was additionally shown to be γδ TCR-dependent, which makes Skint-1 a putative ligand.^109,110^ Mutagenesis of the membrane distal domain of Skint-1 and NMR-studies suggest a putative receptor-interaction surface but a direct interaction with the γδ TCR has not been shown so far.^111^

The case of Btnl/BTNL proteins in mice and humans seems to be, however, much clearer. Btnl1 and 6 form heterodimers and are required for the development of Vγ7^+^ IELs in the gut in a γδ TCR-dependent manner. Likewise, human intestinal Vγ4^+^ TCRs can be activated by the co-expressed Btnl-homologues BTNL3 and BTNL8.^101,112^ TCR-dependent responsiveness to BTNLs seems thus to be evolutionarily conserved. Recently, direct binding as well as the mode of interaction of Btnl/BTNL proteins and the respective γδ TCRs were revealed.^113,114^ The interaction was mediated via the germline-encoded γ-chain HV4 with the involvement of some CDR2 residues and the CFG-domain of Btnl6 and BTNL3, reminiscent of superantigen binding to αβ TCRs.^115^ Other CDRs and the entire δ-chain were not involved but are available for clonally specific ligand binding of e.g. CD1-d or EPCR. This indicates that γδ TCRs are intrinsically able to combine clonal adaptive reactivity and nonclonal innate responsiveness to common ligands. The physiological role of BTNL-responsiveness by γδ TCRs beyond its implication in γδ T-cell development remains to be elucidated. It has, however, been discussed, that it may serve as a signal of normality, keeping the cells ready to respond to cognate antigens under stress conditions.^17,113^

Whether germline-encoded recognition of B7 family-like proteins via the γ-chain HV4 extends also to other γδ TCRs and represents a general principle is unclear. The tissue-specific expression of certain γ-chains would reflect such a broadly applicable mechanism.^116^ Moreover, the recently investigated interaction mode between BTN2A1 and the public Vγ9JP-chain follows the same principle as between BTNL/Btnl proteins and γδ TCRs. In both cases, the HV4 was described as the major mediator of the interaction, suggesting that a binding mode via germline-encoded domains might describe a more general feature of butyrophilin binding by γδ TCRs.

## Discrimination of normal versus stress conditions

**Fig. 1:**
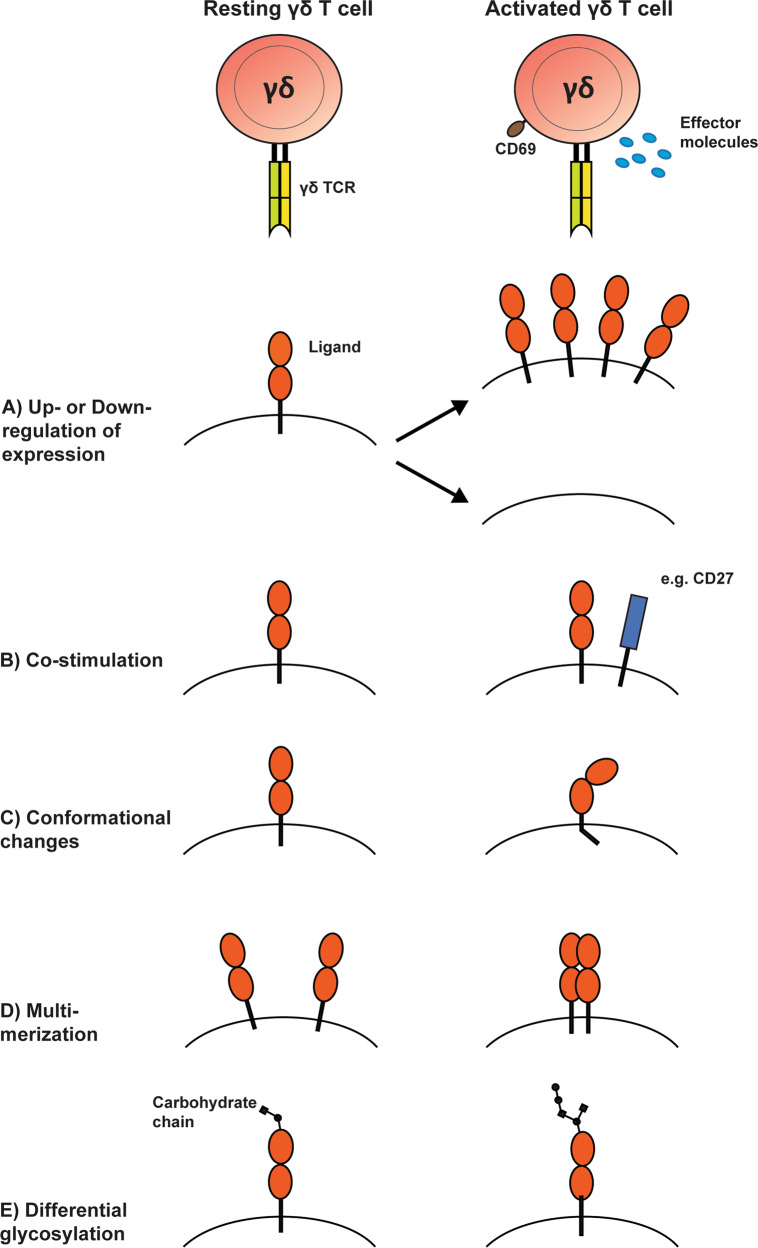
Mechanisms for the discrimination of health and stress conditions via the γδ TCR. **a** The putative γδ TCR ligand might be differentially expressed depending on the stress level of the cell. In the case of stress-induced antigens such as Annexin A2 or MICA/MICB this would mean an upregulation whereas BTNL molecules might be downregulated allowing for γδ TCR activation via the CDR3. **b** Co-stimulatory molecules such as CD27 or JAML might be required for full activation of the γδ TCR and their upregulation might be triggered by stress conditions. **c** Changes in the conformation of the ligand might increase the accessibility of a particular γδ TCR binding domain. BTN3A1 for example undergoes conformational changes upon p-Ag binding. **d** Multimerization or monomerization of the respective ligand can be triggers for γδ TCR as in the case of the HLA-molecule A*24:02. **e** Glycosylation patterns are modified upon infections or tumor development. These changes in post-translational modifications might lead to different outcomes of γδ TCR interaction with the same ligand with different glycan residues on the extracellular domain

Hallmarks of antigen recognition by αβ TCRs are the recognition of pathogen-derived peptides presented by MHC molecules and additional regulation via co-receptors, which allow all together the fine-tuned discrimination of foreign from self. Ligands of γδ TCRs, however, are representing a wide range of largely host-cell-derived molecules, which are believed to be signals of cellular stress (see Table 1). Hence, the question arises of how γδ T cells are able to discriminate normal homeostatic conditions from pathological ones. Especially the direct Ig-like binding of the antigen harbors the potential for autoimmune reactions and thus molecular mechanisms to circumvent this problem must exist (Fig. 1).

One option to regulate activation of the γδ TCR is to induce tissue-specific antigen expression upon stress conditions (Fig. 1a). Examples of antigens differentially expressed at the cell surface are MICA/MICB,^10^ Annexin A2^53^ and EphA2^54^ in humans, which were upregulated upon CMV-infections, hypoxia, heat shock and metabolic reprogramming. In mice, the expression of γδ TCR ligands T10 and T22 was described to be upregulated upon antigenic activation of αβ T cells.^49^ Furthermore, some in vitro studies suggest that the yet unknown antigens for the respective investigated γδ TCRs in Malaria and *Listeria*-infection can already be recognized on target cells prior to any stress stimulus and that the γδ TCR reactivity was increased upon e.g., infection.^117,118^ Besides a transcriptional and translational upregulation of antigen expression, differential subcellular localization of otherwise intracellular γδ TCR ligands might regulate activation. This has been suggested for hMSH2, an otherwise nuclear protein involved in DNA damage repair.^55^

Although this theory of differential expression of γδ TCR ligands seems quite compelling, it does not sufficiently explain all observations made with γδ TCRs investigated for their recognition and binding behavior. In some cases, the expression of the recognized antigen on certain target cells does not induce the respective γδ TCR, e.g., in the case of EPCR,^25^ EphA2^54^ and the MHC-molecules I-E^119^ and HLA-A∗24:02.^23^ Interestingly, although identified as the antigen of a CMV-reactive γδ TCR, the expression of the EPCR is unchanged upon CMV infection. Put together, these observations indicate that either binding of the γδ TCR to its cognate antigen is not always sufficient for its activation and that additional signals or molecular changes in the antigen itself are necessary.

Like αβ T cells which rely on co-receptor and co-stimulatory signals to regulate their response via e.g., CD4/CD8 and CD28, γδ TCRs might also require certain co-receptors for full activation (Fig. 1b). Butyrophilins and the butyrophilin-like molecules Btnl/BTNL might represent candidates for such a regulatory function. As they are binding to germline-encoded regions in the Vγ-chain distinct of the CDR3, they could exert co-receptor functions in addition to the clonotype-specific antigens. However, binding of BTNL3 to human LES TCR (Vγ4Vδ5) was reported to inhibit the interaction with EPCR via the CDR3, indicating that simultaneous interaction with BTNLs and antigen is impossible.^114^ Therefore, BTNLs might not function as classical co-receptors acting in parallel to TCR signaling but rather confer signals of normality before the actual γδ TCR activation. Upon stress conditions such as an infection, BTNLs could be downregulated allowing for the subsequent recognition of stress-induced ligands^17^ (Fig. 1a). In-line with this is the observation that the BTNL expression is altered upon intestinal inflammation and colon cancer in humans and mice.^120,121^ Evidence for this regulatory role of BTNLs in γδ TCR activation is however still missing and it remains therefore purely hypothetical.

A wide range of co-stimulatory molecules required for full TCR-dependent γδ T-cell activation have been described.^122^ These comprise amongst others CD2,^123,124^ CD28,^125,126^ CD27 in the p-Ag-dependent stimulation of Vγ9Vδ2^+^ γδ T cells^127^ and JAML in the activation of murine DETCs.^128^ Besides, CD8α has been shown to increase the reactivity to cancer cells of some Vδ1^+^ TCRs^23,34,129^ and in CMV-positive bone marrow grafts the frequency of CD8^+^ Vδ1^+^ γδ T cells was increased, indicative of a potential co-stimulatory role of CD8 for Vδ1^+^ TCRs.^130^ The exact contribution of all these proposed co-stimulatory molecules to γδ T-cell-mediated immune responses remains to a large extent unclear as they are often only expressed by certain γδ T-cell subsets and functional in vivo studies are missing.

Even more controversial is the role of the Natural-Killer group 2, member D (NKG2D) receptor that was reported to enhance TCR-mediated immune responses of γδ T cells.^55,131,132^ Interestingly, NKG2D-ligands MICA and MICB are also recognized by Vδ1^+^ TCRs and γδ T cells expressing both receptors seem to require signals from both receptors to get activated.^45^ Like other NK-receptors, NKG2D is able to activate γδ T cells without involving the γδ TCR.^133^ It is thus difficult to properly dissect whether the role of NKG2D can be defined as co-stimulatory or whether γδ TCR and NKG2D act sequentially as suggested by Ribot et al.^122^

Besides differential expression and co-stimulation by other surface molecules, intramolecular mechanisms might be involved in the regulation of γδ TCR enabling the distinction of homeostatic and stress conditions. These include e.g., conformational changes that increase the accessibility of domains required for proper γδ TCR activation (Fig. 1c) or post-translational modifications such as glycosylation (Fig. 1e). The p-Ag-mediated conformational changes in BTN3A1 mentioned previously are an example for this.^97,100^ Although no direct binding of the Vγ9Vδ2^+^ TCR to BTN3A1 has been observed so far, this conformational change is required for p-Ag-mediated activation either via BTN3A1 interacting with the CDRs of the TCR or indirectly via a yet unknown molecule. Conformational differences of γδ TCR ligands might also be caused by multimerization as it is the case for the MHC-molecule HLA∗24:02 recognized by the alloreactive Vγ5Vδ1^+^ TCR^23^ (Fig. 1d). Being present as homodimers under regular conditions, malignant transformation seems to induce changes in the spatial organization of HLA∗24:02 at the cell surface so that it occurs as monomers. This restructuring is essential for γδ TCR recognition as fixation of the dimeric form of HLA∗24:02 led to the abrogation of TCR-mediated activation.

The influence of antigen glycosylation on γδ TCR activation has so far not been investigated in detail but some publications suggest that they are relevant for ligand recognition. In fact, changes in surface glycosylation patterns are known to occur in infected or transformed cells.^134,135^ It is thus conceivable that immune receptors recognizing mostly glycosylated cell-surface molecules like γδ TCRs are to some extent reacting to these changes (Fig. 1e). As the investigation of carbohydrates and their effects is difficult with conventional molecular biology techniques, data on the influence of glycosylation on γδ TCR binding and activation is still quite sparse. For example, the recognition of HLA I-E by the murine γδ TCR LBK5 is influenced by changes in the N-glycosylation at position 82 of its α-chain.^119^ Furthermore, recognition of glycosphingolipids by a subset of murine γδ T cells has been reported to be dependent on specific carbohydrate residues but to what extent the γδ TCR is involved in this process has not unambiguously been shown.^136,137^ More recently, a study with soluble γδ TCRs from a patient with Lyme arthritis revealed that binding of the investigated receptors is sensitive to cell-surface digestion of glycans.^138^ Although the actual ligand for the respective γδ TCR was not identified, this hints to an essential role of carbohydrates in the binding mechanism to the target cells. Whether the γδ TCRs in all these studies directly interacts with the respective carbohydrates and whether the influence thereof can be generalized to other γδ TCRs requires further investigation.

Finally, recent data on MR-1-reactive γδ TCRs suggest that also the binding mode of the γδ TCR to its cognate antigen could be of relevance for the degree of activation induced by this interaction.^43^ Some of the investigated human Vδ1^+^ TCRs were interacting with the α3-domain on the side of MR-1 while others were binding from the top to the actual antigen-presenting cleft of MR-1. The γδ TCRs also differed in their capacity to induce intracellular signal transduction. Binding to the α3-domain led to ERK-phosphorylation but no upregulation of the activation marker CD69, whereas interaction with the top-side of MR-1 induced full activation of the employed reporter cells. Although these controversial binding modes where observed in γδ T cells ex vivo from several individuals it is unclear to what extent this applies also to physiological conditions and whether it is a singular or more general phenomenon.

## Concluding remarks

During the past decades, research on γδ TCR ligands revealed a plethora of diverse molecules that are recognized by either unique specific γδ TCRs or by omni-present TCRs with canonical γδ rearrangements such as Vγ9Vδ2. Although some general patterns like involvement of butyrophilins and BTNLs or the recognition of MHC-like molecules can be observed, an overarching concept of what is driving the activation of γδ TCRs is still missing. Thus, in order to broaden our understanding of γδ TCR specificities and their implications in health and disease, unbiased and large-scale screening approaches for further ligands will be required. For αβ TCRs, a multitude of these methods is already established as recently reviewed by Gerber et al.^139^ A prominent example is the expression of MHC-αβ TCR hybrids (MCR) on the surface of a reporter target cell line, which led to the expression of a fluorescent reporter gene construct upon functional αβ TCR binding.^140^ To γδ TCRs however, these approaches cannot be applied since no general restrictive molecule such as the MHC exists or has at least not been identified yet. Therefore, the emerging possibilities of CRISPR-Cas9 technology might be better applicable in this context. With the appropriate readout systems at hand, such as target cell killing or binding of TCR-tetramers, genome-wide CRISPR libraries might allow for a negative selection of those cells where the target for the γδ TCR is not present anymore.

To get a better understanding of γδ TCR activation, the mere identification of ligands on its own will, however, not be sufficient. Especially for host-cell-derived ligands for the γδ TCR, which might be directly recognized without further processing, it is essential to understand the mechanisms by which a discrimination between normal and stress conditions can take place. Besides the differential expression of the respective ligand, co-stimulatory effects by other molecules, multimerization, conformational changes and differential glycosylation of the putative ligand should be considered and investigated more in this context. This should help to reconcile so far contradicting observations in γδ TCR research and help to better understand ligand recognition by the γδ TCR.
